# Lessons learned from the Advancing Maternal Immunization collaboration: identifying evidence gaps for informed respiratory syncytial virus maternal immunization decision-making

**DOI:** 10.12688/gatesopenres.13060.1

**Published:** 2019-09-23

**Authors:** Devin Groman, Deborah Higgins, Sadaf Khan, Evan Simpson, Clint Pecenka, Lauren Newhouse, G. William Letson, Mark Gudmastad, Ranju Baral, Jessica A. Fleming

**Affiliations:** 1 Center for Vaccine Innovation and Access, PATH, Seattle, WA, 98121, USA; 2 Maternal, Newborn, Child Health & Nutrition, PATH, Seattle, WA, 98121, USA

**Keywords:** maternal immunization, vaccine decision-making, gap analysis, respiratory syncytial virus, roadmap

## Abstract

In an increasingly crowded vaccine landscape, global and country decision-makers will require evidence-based and disease-specific information when prioritizing new public health interventions. The Advancing Maternal Immunization collaboration (AMI) was designed to develop a cross-program strategy to advance respiratory syncytial virus (RSV) maternal immunization (MI) availability and accessibility in low- and middle-income countries by completing a comprehensive RSV MI gap analysis and developing an actionable roadmap report. By engaging and coordinating key stakeholders using a web-based communication platform and developing standardized tools, AMI was able to facilitate interaction and consensus between members. This paper describes the methodology used to create and manage AMI’s work. We share lessons learned from our approach to inform other groups conducting similar work requiring cross-sectoral engagement. This approach could be adapted to efficiently conduct gap analyses for other health interventions that require input and coordination across a variety of topic areas, disciplines, geographies, and stakeholders.

## Disclaimer

The views expressed in this article are those of the author(s). Publication in Gates Open Research does not imply endorsement by the Gates Foundation.

## Introduction

Despite significant advances in child survival and well-being, mortality and morbidity rates in the earliest months of life remain high in low-and middle-income countries (LMICs) when infants are particularly vulnerable to certain diseases ^1^. Respiratory syncytial virus (RSV) is a leading cause of infant respiratory infections and hospitalizations worldwide. Of the more than 30 million childhood cases worldwide, RSV causes 3.2 million hospitalizations and 120,000 deaths in children before 5 years of age each year. Half of all RSV-related hospitalizations and deaths occur in the first six months of life, and almost all in LMICs ^2^. Through maternal immunization (MI), mothers can protect their infants and themselves from some diseases by getting vaccinated during pregnancy, with placentally transferred maternal antibodies passively protecting the infant for the first months of life. MI is used safely and effectively in many countries and has a history of success in reducing disease for mothers and infants against pathogens such as tetanus, influenza, and pertussis ^3^. Beyond maternal and neonatal tetanus elimination efforts, however, MI is not widely available in most LMICs ^4, 5^.

Multiple new vaccines designed for MI are in development and maternal RSV vaccines could be the first specifically designed for this purpose to become available in the next several years ^6^. Maternal vaccines, however, present specific challenges for introduction and routine delivery. Expanded Program on Immunization (EPI) and antenatal care (ANC) programs traditionally serve different populations—children and mothers, respectively. Furthermore, these programs are often under-resourced, especially in LMICs. ANC programs in particular may not be set up to focus or dedicate infrastructure to vaccination because vaccination is often offered as just one of a broad array of services or offered at a separate setting than where ANC is provided ^7, 8^. Pregnant women are a traditionally underserved population in immunization; therefore, building consensus on a way forward for RSV MI will require engaging key stakeholders from across immunization and maternal, newborn, and child health (MNCH) communities ^9^.

The pathway from vaccine development to introduction and delivery in LMICs is complex and can vary greatly by vaccine ^10^. Historically, Accelerated Development and Introduction Plans were established to help LMICs navigate this licensure-to-adoption pathway ^11– 13^. In most cases, LMICs look to the World Health Organization (WHO) for guidance on introducing new vaccines, including recommendations from WHO’s Strategic Advisory Group of Experts (SAGE) and eligible countries often procure vaccines with support from Gavi, the Vaccine Alliance ^14, 15^. A key step to enabling timely and informed global and country decisions around introducing a new maternal vaccine is identifying the information requirements for WHO, Gavi, and LMICs.

The Advancing Maternal Immunization collaboration (AMI), led by PATH in coordination with the WHO, was created to identify a viable pathway forward for maternal RSV vaccines and to provide guidance for decision-makers, implementers, researchers, funders, and others to navigate that pathway successfully ^16^. AMI set out to unite thought leaders and technical experts from across the globe and across sectors to assess the full spectrum of requirements for a maternal RSV vaccine to be successful. The WHO’s role as a strategic partner on AMI was key for ensuring the inclusion of decision-making requirements from a global policy perspective. AMI’s process included conducting a RSV MI gap analysis and describing key activities to fill identified gaps in a RSV MI roadmap. With several maternal vaccines on the horizon, we built our approach to be flexible and adaptable to support the unique challenges presented by maternal RSV vaccines while also informing the needs of a broader MI platform ^6^.

This paper describes the methodology used to create and manage AMI. We explain the process used and tools developed to minimize the time and resources required to conduct the gap analysis and develop the roadmap. We share lessons learned from our approach to inform other groups conducting similar work with maternal vaccines or other disease prevention efforts requiring cross-sectoral engagement.

## Methods

**Figure 1.**
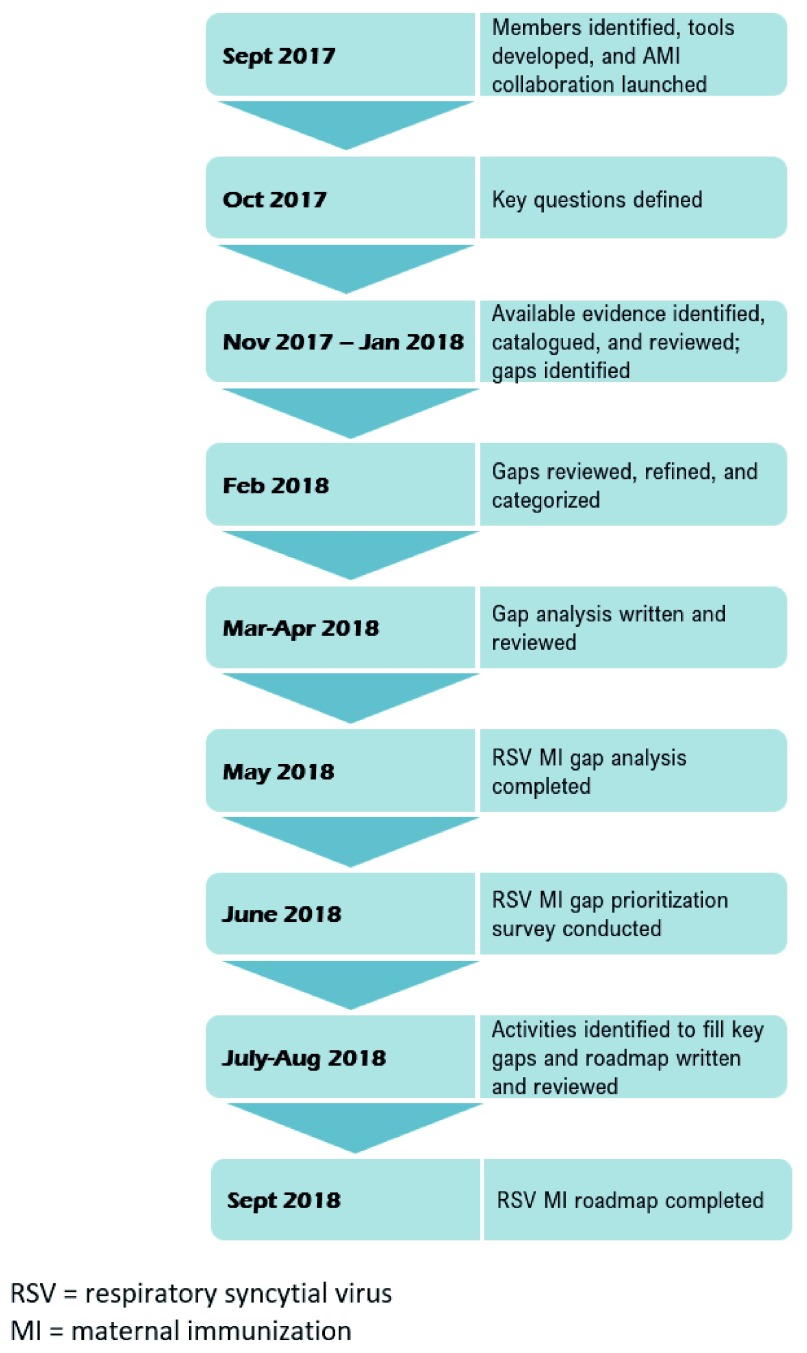
Advancing Maternal Immunization (AMI) timeline to produce RSV MI gap analysis report and roadmap.

AMI’s mandate was to conduct a comprehensive gap analysis of RSV MI, organize findings into a gap analysis report, and develop an actionable roadmap report within 12 months ( Figure 1). For the gap analysis, we identified information needs (termed “key questions”), catalogued existing information, and identified gaps in evidence. The roadmap followed from this effort by recommending activities to fill the gaps outlined in the gap analysis. The following section describes the methodology we used to design and launch AMI; identify and collaborate with key stakeholders; and develop tools to facilitate research, communication, and report writing.

### Designing AMI’s structure and membership

AMI included 62 diverse technical and programmatic experts in public health from over 25 organizations across 14 countries. AMI members were identified based on primary and secondary selection criteria. Primary selection criteria included experience, training, and expertise in immunization, maternal child health care, and/or vaccine decision-making relevant to LMIC contexts. Secondary selection criteria included gender, geography, and technical skills relevant to the particular AMI group joined. Members’ combined expertise reflected multiple disciplines across the epidemiology of RSV and respiratory infections, vaccine development, health economics and financing, and vaccine introduction and delivery specific to LMICs. We sought equal representation from individuals with MNCH and vaccine expertise in the groups described below and included representation from the WHO throughout.

**Figure 2.**
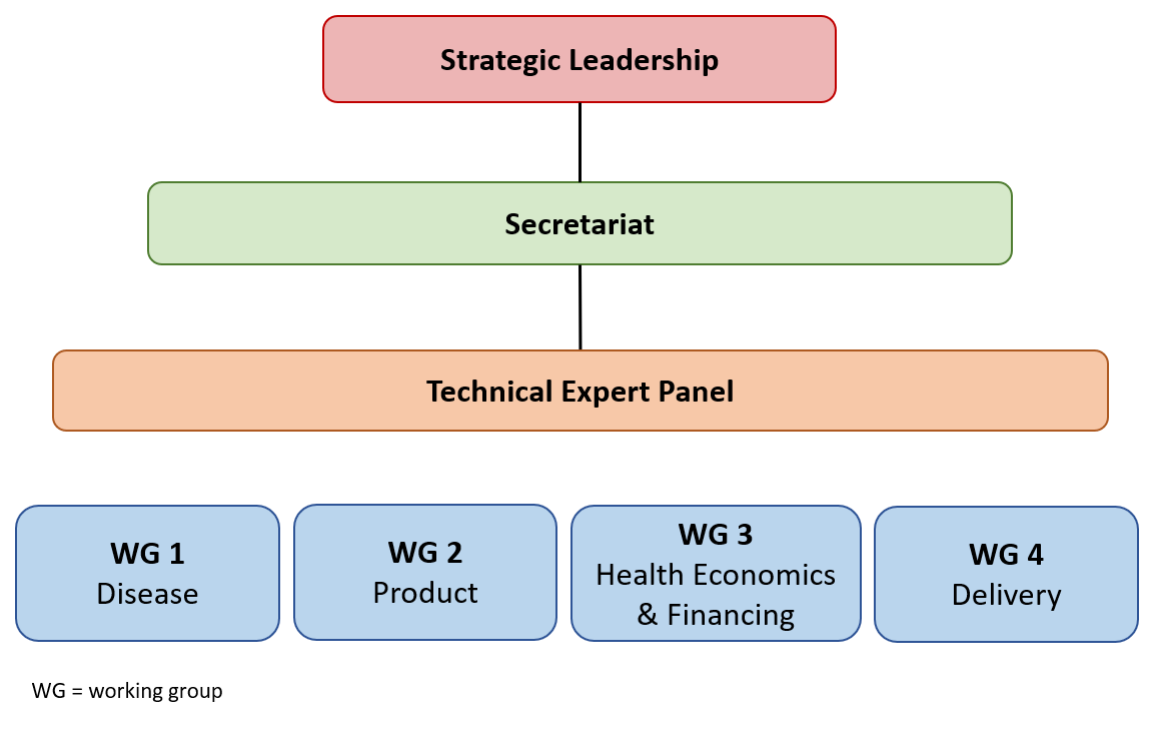
The Advancing Maternal Immunization collaboration structure.

AMI’s structure comprised four distinct groups with unique roles: Strategic Leadership (SL), Secretariat, Technical Expert Panel (TEP), and Working Groups (WGs) ( Figure 2).

The SL provided strategic direction for AMI and comprised five leaders from the WHO and PATH with expertise in vaccine development, vaccine delivery, and MNCH. The ten-member TEP defined the information required for vaccine decision-making and introduction, provided expert opinion on gap priorities, and reviewed documents produced by AMI members. TEP expertise included vaccine licensure and WHO prequalification (PQ); MI and RSV; MNCH; global vaccine policy and financing; LMIC public health decision-making; and program implementation. The nine members of the Secretariat, housed at PATH, were responsible for providing day-to-day support to AMI through technical and administrative oversight, coordinating member inputs, finalizing outputs, and creating communications materials.

WGs included 40 technical experts from academia and research organizations, policymakers in LMICs, and practitioners from across MNCH, immunization, and other relevant sectors. WG members conducted background research on specific topics, summarized existing evidence, and identified gaps in the evidence and conditions relative to RSV MI decision-making and introduction. Table 1 shows the four WG topic areas. These topics were chosen to reflect the broad categories outlined in the 2015 WHO SAGE “Guidance for the development of evidence-based vaccination-related recommendations” ^17^. Each WG had eight members except for the delivery WG, which had sixteen members because it covered a broader range of topics. Several AMI members participated in multiple WGs.

**Table 1.** Advancing Maternal Immunization working groups, member expertise, and review focus.

Working group topic	Number of members	Member expertise	Review focus
**Disease**	8	• Epidemiology of disease • Clinical disease characteristics • Current disease control and prevention • Laboratory surveillance considerations • RSV long-term sequelae	• Respiratory syncytial virus (RSV) burden of disease in infancy and pregnancy • Disease factors pertinent to informing the potential use of a maternal RSV vaccine in low- and middle-income countries (LMICs) • RSV long-term sequelae
**Product**	8	• Vaccine and immunization characteristics • Respiratory diseases, especially RSV • Clinical evaluation of vaccines, especially maternal vaccines • Vaccine safety • Vaccine registration • Vaccine programmatic suitability and prequalification • Manufacturer commercialization and supply for LMICs	• Key immune parameters and clinical evaluations of maternal RSV vaccines to inform decisions around use in immunization programs • Vaccine safety and pharmacovigilance • Meeting LMIC vaccine supply needs
**Health** **economics** **and** **financing**	8	• Global health and financing policy • LMIC policy expertise in Expanded Program on Immunization, maternal health/antenatal care/ neonatal health • RSV and other respiratory diseases • Knowledge of maternal immunization • Economic modeling, cost of illness, and cost of delivery	• RSV cost of illness • Costs of intervention delivery • Demand, impact, and cost-effectiveness • Financing and budget impacts of the vaccine in LMIC contexts
**Delivery**	16	• Maternal and newborn health • Social and behavior change communication • LMIC clinical/field • Health systems • Integration program implementation • Policy and advocacy • Healthcare seeking behaviors • Capacity building • Immunization delivery	• Health systems and patient, provider, and community factors relevant to maternal RSV vaccine introduction and uptake in LMICs, including policy considerations • Key stakeholder awareness, perceptions, and acceptability • Vaccine logistics and supply chain • Care-seeking and care-provision in pregnancy • Health systems • Ethical, cultural, and gender issues relevant to vaccine uptake

Individuals from the Secretariat with relevant expertise served as WG coordinators (WGCs). WGCs were responsible for organizing and managing their WG members and overseeing evidence reviews. Within each WG, individuals were identified to serve as ‘question leads’ to manage the compilation and synthesis of existing evidence, information, or relevant conditions for a specific question(s).

### Conducting a gap analysis and creating a roadmap

For the gap analysis report, the Secretariat built upon published guidelines and existing tools and developed new tools and data collection templates to guide and standardize information from reviews ^17– 20^. To frame key questions and refine the specific information needs, the Secretariat drafted an initial list of questions based on the aforementioned WHO SAGE guidance document ^17^. Building on and revising the draft list, TEP members identified key questions to inform evidence-based global and country stakeholders’ decisions around RSV MI. The Secretariat assigned questions to a WG according to topic area.

To standardize the information collected for each key question and promote consistency among reviewers, the Secretariat developed a question ‘framework’ template (see 21, Appendix 2). Question leads completed the frameworks through a combination of desk research, expert interviews, and expert opinion. In consultation with AMI members and external experts, question leads evaluated the available evidence and made a judgement as to the degree to which it was sufficient to inform decision-making and RSV MI introduction in a LMIC context based on generalizability, context and location, consistency, overall quality, and relevant limitations or biases. If the evidence was considered *sufficient,* the question was characterized as having no gaps. If the evidence was considered *insufficient*, question leads identified gaps in the evidence that were essential for decision-making and/or introduction and described any relevant ongoing work to fill the gap.

Within a given WG, members with relevant expertise and interest self-selected to serve as first-line reviewers of each question and provided feedback to question leads on the question frameworks and specific gaps identified. Once a framework was completed, question leads disseminated it to relevant reviewers (including the TEP and SL) to check for accuracy and completeness. As necessary, question leads sought additional *ad hoc* input from external parties.

Once completed, the Secretariat identified overlaps in information and created a list of gaps by topic. In some cases, we maintained similar gaps identified by multiple WGs to preserve the unique perspective of each group and to highlight their crosscutting nature. The Secretariat sorted gaps into one of four categories based on their perceived importance for decision-making and introduction in LMICs and their uniqueness to MI, described in Table 2. WG members reviewed the full list of gaps for accuracy and agreement on categorization.

**Table 2.** Gap categories and their definitions.

Gap category	Definition
**Essential and specific to maternal** **immunization (MI)**	A gap in information or conditions that is unique to MI and that must be addressed for MI decision-making and/or introduction to move forward.
**Essential across immunizations**	A gap in information or conditions that is generally applicable across vaccines and that must be addressed for MI decision-making and/or introduction to move forward.
**Non-essential but supportive**	A gap in information or conditions that, if addressed, could strengthen or accelerate MI decision-making and/or introduction, but is not required to move forward.
**Non-essential and peripheral**	A gap in information or conditions that may be of interest, but does not need to be addressed to advance, strengthen, or accelerate MI decision-making and/or introduction.

After reviewing 99 question frameworks, AMI members identified 57 individual gaps. The full list of gaps and their categories were described in the RSV MI gap analysis report, completed in May 2018 ^21^. This report identified essential gaps in information and conditions needed to support RSV MI decision-making and delivery in LMICs. It identified relevant work already completed or underway and recommended areas where more effort was needed.

Gaps from the first three categories were used to identify activities required for RSV MI decision-making and delivery to be successful and were later outlined in the RSV MI roadmap ^22^. Gaps were subsequently prioritized through an online survey developed by the Secretariat. The survey was sent to 175 people, including AMI members and other technical experts identified by AMI members, and had a 40% response rate. Respondents were asked to rate gaps according to their importance to RSV MI global and country decision-making, as well as implementation in LMICs.

Based on the RSV MI gap analysis and the online survey, the Secretariat compiled the results and identified the activities necessary to fill prioritized gaps for inclusion in the RSV MI roadmap. Activities were placed on a projected timeline across the licensure-to-adoption pathway and divided into near-term and mid-to-long-term activities. Activities were classified as near-term or long-term based on 1) implications for an imminent decision point such as WHO PQ or SAGE recommendation; 2) the length of time needed to plan and accomplish the activity; 3) the reliance of other key activities on the results or outcomes of the activity; and 4) the need for continuity with ongoing or recent activities. After review from AMI members, the Secretariat (with guidance from the SL) made final determinations on the activities included in the roadmap and their prioritization. AMI completed the roadmap in September 2018, which outlined activities required for RSV MI decision-making and delivery to be successful ^22^. The RSV MI roadmap clarified why certain activities were needed (for instance, which global or country decision-makers the activities would inform) and flagged where new activities could complement or build upon completed or ongoing work.

The RSV MI gap analysis and RSV MI roadmap are available online ^21, 22^ and findings will be published separately.

### Facilitating global communication

Despite the size and scope of this effort, no in-person meetings were planned with all AMI members due to members being based in numerous locations around the world, a limited budget, and a need to maximize time efficiency. In lieu of this, the Secretariat held regular conference calls with the SL and TEP, and WGCs held smaller teleconferences with their WGs. In addition, the Secretariat created a web-based platform (using IGLOO™ Software) termed the “Knowledge Center” as a virtual members-only meeting space. The Secretariat chose this platform based on specific functions including ease of use; ability to collect, edit, and organize content effectively; and availability of a variety of interactive tools to support communication and relationship building among members. The Secretariat delivered several one-hour trainings by teleconference to orient AMI members to the platform and its key features.

Each WG had a designated workspace on the Knowledge Center to contact AMI members within their group, pose questions, and monitor group progress. Members also used the Knowledge Center to contribute to question frameworks, including direct editing, adding comments, and communicating with question leads directly, as necessary. Members had access to all WG spaces and were encouraged to explore and engage with other WGs working on topics within their area of expertise.

The Secretariat met in person regularly to discuss each WG’s progress, coordinate across WGs, and identify when other AMI members (including the TEP or SL) should be consulted. When necessary, WGCs organized teleconferences with WG members to answer questions, reach consensus, and connect members working on similar questions to reduce duplicative efforts. For members with challenges in accessing the internet, the Secretariat provided alternative methods to engage, including one-on-one phone calls or e-mail communication.

## Discussion

AMI members reviewed available literature and completed the gap analysis and roadmap reports within 12 months, incorporating knowledge and expertise from global experts across immunization and MNCH disciplines. While the process we used was relatively efficient, challenges to implementation existed. The deliberate structure of AMI’s member groups, the Secretariat promoting communication through the web-based Knowledge Center, and use of standardized tools, however, enabled work to progress efficiently. Below we outline the advantages, challenges, best practices, and lessons learned from the approach we used to conduct the gap analysis and produce the roadmap.

### Membership

Inclusion of members with diverse expertise across immunization and MNCH disciplines enabled AMI to produce work products that addressed all topics recommended by the WHO to move a vaccine toward a SAGE recommendation and PQ with broad consensus across key stakeholders in MI ^17^. To minimize the timeframe required, systematic reviews were not conducted for all topics. Instead, AMI’s efforts leveraged the cumulative expertise of its members and outside experts to validate findings. To take advantage of key expertise and existing efforts, the Secretariat also involved authors of complementary reviews and guidance documents ^18, 20^. This cross-program collaboration enhanced awareness and knowledge of diverse bodies of work.

One limitation of our approach was our reliance on reviews and recommendations by subject matter experts, which may have created potential for missing information and/or introducing biases. Topics not considered critical for global and country decision-making by a larger audience may have been included, despite efforts to focus the review on knowledge gaps that were most likely to delay decision-making if left unfilled. Similarly, while iterative reviews encouraged complete and unbiased results, the Secretariat made decisions around final content based on guidance from the SL and TEP.

### Structure

AMI’s organizational structure was strategically designed to include a balance of skills across groups, clearly defined roles, and representation across immunization and MNCH disciplines. The Secretariat and WGs included both technical expertise and administrative coordination to facilitate efficient collaboration and create quality products. Having WHO representation on the SL, TEP, and WGs ensured that global policy perspectives were included in each of our research areas. Including decision-makers from LMICs and experts with SAGE and PQ experience on the TEP facilitated the incorporation of best practices from their learnings. Key roles such as the WGC and question lead allowed multiple work streams to progress in parallel. Question leads reviewed available evidence within two months with support from WGCs, who facilitated collaboration between and among WGs and communicated with the Secretariat to engage SL and TEP members, as necessary. By meeting regularly to discuss WG progress, WGCs reduced the likelihood of duplication across topics and raised issues or concerns on an ongoing basis. Both efforts reduced the amount of time required to conduct the work.

Due to the size of the WGs and the roles individuals assumed within their group, member engagement throughout the gap analysis process varied greatly. Some members experienced poor internet access and were unable to reliably connect to online tools, requiring them to provide input through email or phone communication with their WGC. In addition, because AMI members were in large part donating their time to this effort, competing priorities created delays at times and may have reduced overall engagement for some members. The Secretariat provided a variety of avenues for participation including e-mail and one-on-one phone conversations to accommodate these impediments and provided multiple opportunities to contribute with frequent follow-ups. In addition, many SL, TEP, and WG members acknowledged that conducting the RSV MI gap analysis and developing the roadmap were important for their own fields of work and/or study, which provided personal incentive to complete the work in a timely fashion.

### Communication strategy

Using the web-based Knowledge Center as the primary means for communication between AMI members came with advantages and disadvantages. As an advantage, the Knowledge Center facilitated connecting and coordinating work among members in a low-cost, user-friendly way without having to rely on in-person or teleconference meetings. As a centralized location for all information, the Secretariat found it a useful tool to promote transparency within AMI and throughout the gap analysis process. AMI members had access to all WG workspaces and all comments and edits could be viewed during the review process. This open access approach facilitated collaboration and information sharing among colleagues and across WGs. The Knowledge Center further allowed members to archive their work and resources, while document version control ensured transparent writing and review processes.

The implementation of this Knowledge Center, however, required additional staff time for the initial build of the platform and to maintain it as a useful resource. Prior to launching the Knowledge Center, AMI members required training on its use. This one-hour online training was an extra required step in the process and was not attended by all members. Knowledge Center use also varied by member and WG. As a result, some members engaged with the Knowledge Center more than others. Also, during intensive review periods, some members submitted edits outside of the Knowledge Center, making consolidation of sometimes-contradictory comments challenging. These instances were resolved through further discussion with WG members and vetting through the TEP and SL.

### Tools

The Secretariat aimed to introduce as much efficiency in our approach as possible by adapting existing tools, when available, and developing new templates and tools such as question frameworks and gap category definitions. Utilizing the question frameworks helped ensure that question leads and WG members reviewed information uniformly, allowing the Secretariat to streamline findings for the final gap analysis report. Delegating work to fill in the question frameworks to question leads actively engaged members and allowed work to be conducted in parallel, ultimately reducing the time required for completion. Similarly, reviews of individual question frameworks were accelerated because information was presented uniformly across frameworks. This facilitated identifying additional gaps or recognizing work that was ongoing to fill the gaps.

A continuous challenge was our ability to control the risk of including individual biases in the final work products. While conducting the gap analysis, many gaps were identified, but not all of them were considered of equal importance to decision-making and introduction in LMICs. Instead of deleting low-priority gaps, categorizing them based on their perceived criticality offered flexibility to include gaps that were peripheral to the intended scope of the gap analysis, but were considered by the larger group to be relevant research questions. Using this gap categorization, the Secretariat sought to represent the work of its members in a balanced way and to maintain equipoise.

## Conclusion

AMI produced a comprehensive RSV MI gap analysis and roadmap by engaging and coordinating key stakeholders with expertise and experience along the vaccine licensure-to-adoption pathway, including both vaccine and MNCH perspectives. This approach may serve as a framework for future collaborations where systematic analyses are not possible. Groups such as the WHO’s Accelerate Cervical Cancer Elimination Initiative have already recognized some of the tools developed by AMI as useful for their work and used them as guides during a recent meeting ^23^. Taking into account the best practices and lessons learned from our approach, other organizations are encouraged to adapt it for their specific needs.

In an increasingly crowded vaccine landscape, global and country decision-makers will require evidence-based information when making decisions about prioritizing new public health interventions. Need is increasing for cross-program collaboration and intersectoral work that engages diverse expertise to consider novel approaches that facilitate working together. This is particularly evident for innovations that require modifications to existing systems and services or a shift in perspective, such as maternal vaccines. AMI’s process of defining information needed and prioritizing a way forward is one example of how key stakeholders in the field can be engaged to efficiently contribute to charting the licensure-to-adoption pathway while working within limitations of time and financing. The AMI model could be adapted to efficiently conduct gap analyses for other health interventions that require input and coordination across a variety of topic areas and stakeholder expertise.

## Data availability

No data is associated with this article.
