# Supplementary material for: Lessons learned from the Advancing Maternal Immunization collaboration: identifying evidence gaps for informed respiratory syncytial virus maternal immunization decision-making
Source: Gates Open Res. 2019 Sep 23;3:1544. [Version 1] doi: 10.12688/gatesopenres.13060.1 (PMC6978845; doi:10.12688/gatesopenres.13060.1)
Supplement: Peer review history

# Peer review: Reviewer response for version 1

**DOI:** 10.21956/gatesopenres.14190.r28464

**Published:** 2020-01-22

**Authors:** Christopher J. Gill

**Affiliations:** 1 Department of Global Health, Boston University School of Public Health, Boston, MA, USA

The authors describe lessons learned from a collaborative exercise aimed at identifying gaps in knowledge needed to reach decisions about the possible future introduction of maternal RSV vaccinations. Potential readers should note that this article's scope is limited to describing the process by which this process was conducted, but do not discuss the results of the deliberations per se. Thus, the value in the piece is in documenting an approach to collective decision making across multiple geographically disbursed members. 

 I have no major concerns about the article, though I had some minor points for the authors to consider.

1. Abbreviations: PATH should be spelled out. 
2. In referring to the BOD work by Shi in ref 2 ^1^, please just note that the data that fed into that meta analysis were largely from hospitals. The authors openly acknowledge that limitation. As a result, the true BOD of RSV may be considerably higher than the published estimates since these miss the community burden of disease.

I confirm that I have read this submission and believe that I have an appropriate level of expertise to confirm that it is of an acceptable scientific standard.

## References

[1] : Global, regional, and national disease burden estimates of acute lower respiratory infections due to respiratory syncytial virus in young children in 2015: a systematic review and modelling study. *The Lancet*.2017;390(10098) : 10.1016/S0140-6736(17)30938-8 946-958 10.1016/S0140-6736(17)30938-8 PMC559224828689664

# Peer review: Reviewer response for version 1

**DOI:** 10.21956/gatesopenres.14190.r27908

**Published:** 2019-09-27

**Authors:** Ralph A. Tripp

**Affiliations:** 1 Department of Infectious Disease, University of Georgia, Athens, GA, USA

Is the rationale for the Open Letter provided in sufficient detail?

- Yes, AMI produced a comprehensive RSV MI gap analysis and roadmap that was facilitated by engaging stakeholders with KOLs and their expertise. It is clear that an MI platform that normalizes vaccination practice and is supported by (continuing) education and communication is vital.

Does the article adequately reference differing views and opinions?

- Yes.

Are all factual statements correct, and are statements and arguments made adequately supported by citations?

- Yes, the statements and arguments were supported. The article takes into account the best practices and lessons learned. It is clear that a well-informed vaccine supply strategy is going to be key in achieving widespread licensure and availability in LMICs.

Is the Open Letter written in accessible language?

- Yes.

Where applicable, are recommendations and next steps explained clearly for others to follow?

- The recommendations/next steps are explained clearly. It was noted that the Secretariat was responsible for sorting the gaps into categories based on their perceived importance for decision-making and their uniqueness to MI. The tables in the report might consider informing on the gaps in information that are unique to MI and the decision-making process specific to an LMIC market/region, and how an RSV vaccine is going to be delivered to that market/region.
- Perhaps the report might also note/summarize what additional ad hoc input from external parties was considered in the decisions.
- The concerns about vaccine safety given during pregnancy are a known barrier among the public and healthcare providers. Given the history of RSV vaccines, this and related concerns (e.g. vaccine effectiveness) might be addressed separately (own paragraph) when discussing MI.

I confirm that I have read this submission and believe that I have an appropriate level of expertise to confirm that it is of an acceptable scientific standard.
